# Mechanistic insights into the role of herpes simplex virus 1 in Alzheimer’s disease

**DOI:** 10.3389/fnagi.2023.1245904

**Published:** 2023-09-07

**Authors:** Shu Feng, Yongzhen Liu, Yu Zhou, Zhenfeng Shu, Zhuxi Cheng, Charles Brenner, Pinghui Feng

**Affiliations:** ^1^Department of Diabetes and Cancer Metabolism, City of Hope National Medical Center, Duarte, CA, United States; ^2^Section of Infection and Immunity, Herman Ostrow School of Dentistry, Norris Comprehensive Cancer Center, University of Southern California, Los Angeles, CA, United States; ^3^International Department, Beijing Bayi School, Beijing, China; ^4^Department of Molecular Microbiology and Immunology, Norris Comprehensive Cancer Center, Los Angeles, CA, United States

**Keywords:** Alzheimer’s disease, HSV-1, genetic risk genes, innate immunity, metabolism

## Abstract

Alzheimer’s Disease (AD) is an aging-associated neurodegenerative disorder, threatening millions of people worldwide. The onset and progression of AD can be accelerated by environmental risk factors, such as bacterial and viral infections. Human herpesviruses are ubiquitous infectious agents that underpin numerous inflammatory disorders including neurodegenerative diseases. Published studies concerning human herpesviruses in AD imply an active role HSV-1 in the pathogenesis of AD. This review will summarize the current understanding of HSV-1 infection in AD and highlight some barriers to advance this emerging field.

## 1. Introduction

**Figure 1:**
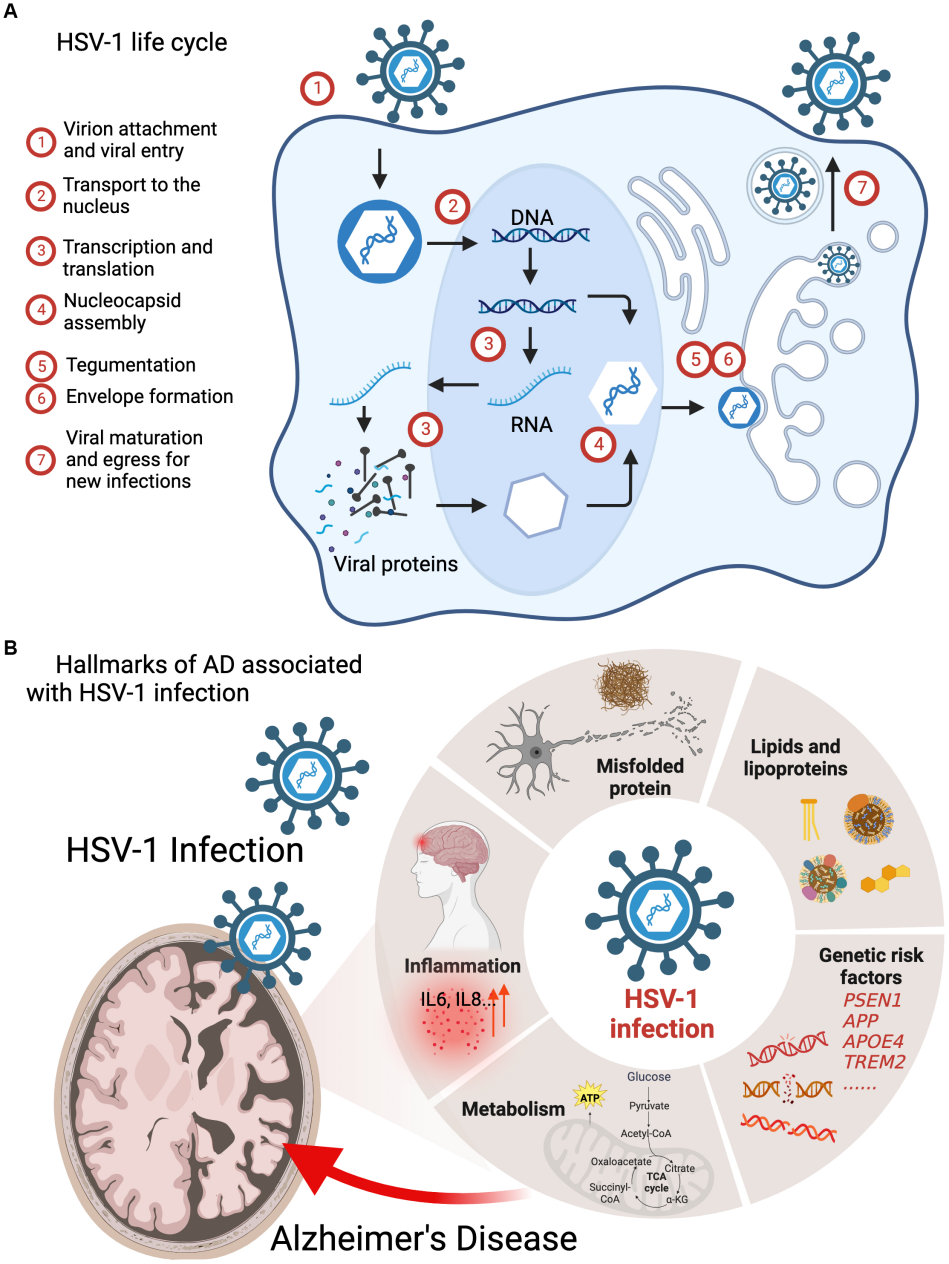
HSV-1 infection interferes the pathogenic processes of AD. **(A)** HSV-1 life cycle: (1) virion attachment and viral entry; (2) transport to the nucleus; (3) transcription and translation; (4) nucleocapsid assembly; (5) tegumentation; (6) envelope formation; (7) viral maturation and egress for new infections. **(B)** Hallmarks of AD including genetic risk factors, amyloid plaques, hyperphosphorylated tau, inflammation and metabolism are associated with HSV-1 infection.

Viral infections represent a major challenge to public health, as evidenced by the COVID-19 pandemic (Morens and Fauci, 2020; Hu et al., 2021). Herpesviruses are ubiquitous human pathogens associated with a broad spectrum of acute and chronic diseases, including genital herpes, lymphoma, Kaposi’s sarcoma, encephalitis, stomach and nasopharyngeal cancer, coronary heart diseases, atherosclerosis, and neurodegeneration (Ashley and Wald, 1999; Whitley and Roizman, 2001; Leinonen and Saikku, 2002; Gupta et al., 2007; Dittmer and Damania, 2016; Readhead et al., 2018; Marcocci et al., 2020; Casanova and Abel, 2021). Herpes simplex virus 1 (HSV-1), also known as human herpesvirus 1 (HHV-1), establishes a life-long persistent infection in humans (Davison, 2010; Wilson and Mohr, 2012; Grinde, 2013), particularly as latent infection in neurons. HSV-1 infection results in acute lytic replication in oral epithelium and undergoes latent infection primarily in trigeminal ganglia. HSV-1 can transport through synaptically connected neurons to the central nervous system (CNS), and latent infection in the brain stem was reported in mice (Doll et al., 2019; Zhang S. et al., 2022). In addition, HSV-1 infection alters the integrity and permeability of the blood–brain barrier (BBB), which allows other infectious agents to transport into the brain parenchyma, exacerbating infection and provoking inflammation (Sweeney et al., 2018; Liu H. et al., 2019). Recent estimate of HSV-1 prevalence in the US, reported by the Centers for Disease Control and Prevention, was approximately 47.8% in the general population and increased linearly with age (McQuillan et al., 2018). Furthermore, aging and aging-associated stress conditions can trigger HSV-1 reactivation in infected cells (Stowe et al., 2012). As an obligate intracellular pathogen, HSV-1 replication relies on cellular machinery to produce viral structural components, assemble virion particles, and to support maturation and egress that yield infectious viral progeny dissemination and infection (Figure 1A; Mettenleiter, 2002). HSV-1 virion consists of a linear double-stranded DNA genome wrapped by an icosahedral capsid, which is encircled by both tegument and viral envelope (Bauer et al., 2013; Liu Y. T. et al., 2019). During a series of viral replication steps, the intimate interactions between HSV-1 and neuronal cells alter cellular homeostasis pathways, such as DNA damage and repair, metabolism, and immune response, which increase the risk of neuronal damage and cognitive impairment, instigating or exacerbating the aging-associated neurodegeneration underpinning Alzheimer’s disease (AD) (Conrady et al., 2010; Katan et al., 2013; Doll et al., 2019; Laval and Enquist, 2021; Sawtell and Thompson, 2021). Accruing evidence has suggested that microbial infections accelerate the pathogenesis of AD, and HSV-1 is the most highly suspected culprit (Heneka et al., 2015a; Ezzat et al., 2019). The presence of HSV-1 in the brain of AD patients was unambiguously confirmed by standard molecular detection methods such as PCR and *in situ* hybridization. Moreover, big data mining and association analysis provide molecular insights into how HSV-1 promotes neurodegeneration and AD progression (Readhead et al., 2018).

Alzheimer’s Disease, the most common form of dementia, is a debilitating neurodegenerative disorder resulting in cognitive impairments, and it is incurable (Knopman et al., 2021). From 1990 to 2019, the incidence of AD increased about 2.5-fold to 7.24 million cases worldwide. In addition, the estimated number of AD cases had reached 13.6 million in East Asia, 7.3 million in Western Europe, and 5.4 million in North America (Li et al., 2022). This rapidly increasing prevalence of AD highlights the urgency and importance of investigating core mechanisms of and developing innovative therapeutics for AD. The major pathology of AD is the accumulative senile plaques (Aβ_42_ aggregate) and neurofibrillary tangles, that induce neuronal dysfunction and eventual cell death. Over the past decade, key biological features of the AD brain have been continuously identified, including neuroinflammation, metabolic dysregulation (Heppner et al., 2015), mitochondria dysfunction, oxidative stress, proteostasis perturbation and lipid abnormalities (Yin, 2023). Given the underlying causes and the pathological prognosis of AD are multifactorial, more risk factors, such as genetic mutations (Satizabal et al., 2019; Tasaki et al., 2019), traumatic brain injury (TBI) (Gardner and Yaffe, 2015), Down syndrome (Hartley et al., 2015), proinflammatory immune stimulation (Golde, 2019), and microbial infections (Readhead et al., 2018; Dominy et al., 2019) are emerging as prominent risk factors for neurodegeneration underpinning AD. Examining their contribution will better our understanding in the molecular pathogenesis of AD. Despite extensive clinical trials for AD treatment, no success has been reported until the most recent anti-Aβ antibody trial and a tau-targeting antisense oligonucleotide (MAPTRx) trial that showed promising response in some patients with significant side effect in others (Mummery et al., 2023; van Dyck et al., 2023). Thus, these observations suggest that current understanding of AD pathogenesis is inadequate.

There is an increasing appreciation of microbial infection in neurological disorders and chronic neurodegenerative diseases (Duggan et al., 2020; Athanasiou et al., 2022). Epstein–Barr virus (EBV) infection was found to greatly increase the risk of multiple sclerosis (Lanz et al., 2022). Influenza and pneumonia are associated with five neurodegenerative diseases, including AD, amyotrophic lateral sclerosis (ALS), generalized dementia (DEM), vascular dementia (VAS) and Parkinson’s disease (PD) (Levine et al., 2023). HSV-1 and HSV-2 infection accelerate the development of AD and AD-like neurodegeneration *via* instigating amyloid aggregation and neuroinflammation (Berger and Houff, 2008; Kristen et al., 2015; Zhang Y. et al., 2022). Moreover, persistent viral infection, particularly that of HSV-1 and -2, usually occurs early in life and far prior to the onset or diagnosis of neurodegenerative diseases (Mangold and Szpara, 2019; Zhu and Viejo-Borbolla, 2021). The reactivation of latently infected viruses during aging may increase the risk of brain diseases. As such, it is of importance to summarize current findings of how HSV-1 interacts with key components that drive the progression of AD. In this review, we summarize the recent advances concerning the roles of HSV-1 in AD pathogenesis, with focus on genetic risk factors, AD pathologies, inflammation, and metabolism (Figure 1B).

## 2. APOE4 and other genetic risk factors are not only associated with AD, but with HSV-1 infection

The Apolipoprotein E (APOE) gene exists in three common isoforms, i.e., APOE2, APOE3 and APOE4. These apolipoproteins E are the building blocks of many lipoproteins and can assist with their transport across the plasma membrane (Liu et al., 2013). The APOE4 isoform is a primary genetic risk factor of AD, which has been shown in the population-based cohort studies (Farrer et al., 1997; Linard et al., 2021) and genome-wide meta-genomic analysis (Lambert et al., 2009). The frequency of HSV-1 reactivation is corelated with the development of AD among APOE4 carriers from a cohort study (Linard et al., 2020). As reported, a 3-fold increase of AD incidence, calculated with adjusted hazard ratio, was observed in the patients with positive immunoglobulin M (IgM) or elevated levels of IgG against HSV-1. In the study using APOE4 knockout mice, Burgos et al. (2006) detected significant lower HSV-1 genomes in the nervous system than that in the wildtype mice, suggesting that APOE4 facilitates HSV-1 invasion and latency establishment in the brain. Later, by examining key steps of HSV-1 lytic replication, Liu et al. (2023) surprisingly demonstrated that APOE4 protein inhibits HSV-1 attachment but not viral entry, replication, assembly, or intracellular transportation of lytic replication, which may contribute to the enhanced latency. Paradoxically, APOE4 can be incorporated into HSV-1 virions to promote viral release from cell membrane (Liu et al., 2023). These findings indicate that APOE4 has distinct function in various steps of HSV-1 lytic replication, although the discrepancies remain unexplained. Aligning HSV-1-encoded proteins to human proteome identified viral protein sequences that mimic several human proteins (APOE4, CR1, PICALM, BACE1, BACE2 and gamma-secretase components), which are expressed by major AD genetic risk loci (Carter, 2010a). These host proteins were found to play regulatory roles in HSV-1 infection and replication (Table 1; Harris and Harris, 2018; Readhead et al., 2018), For example, PICALM regulates the transmembrane protein cation-independent mannose-6-phosphate IGF2 receptor (IGF2R) that is responsible for HSV-1 entry and cell-to-cell transmission, thus impacting HSV-1 replication and spread in the brain (Carter, 2011). Together, these findings highlight the synergistic interaction between human genetic makeup and microbial infection in shaping the AD pathogenesis. Genetic variants linked to the immune system, e.g., ADAM17, also known as TNF-α-converting enzyme and TNIP1, and LUBAC (involved in NLRP3 inflammasome activation) participate in immune signaling, such as TNF-α signaling pathways (Bellenguez et al., 2022). However, how HSV-1 infection interacts with these inflammatory genes is poorly understood. Perhaps, this may represent a sprouting area that human genetic variation and environmental factors synergize to accelerate the aging-associated neurodegeneration, leading to diverse forms of dementia including AD.

**Table 1:** List of major Alzheimer’s disease risk genes and connections to HSV-1 infection.

Genetic risk factors	Connection to HSV-1	References
Apolipoprotein E isoform 4 (APOE4)	Facilitate HSV-1 invasion and latency establishment; enhance HSV-1 detachment from the cell membrane	Linard et al. (2020), Burgos et al. (2006), Liu et al. (2023), Carter (2010a), Harris and Harris (2018)
Clusterin (CLU)	Influence endosomal routing pathways used by HSV-1 trafficking; play a role in the complement system interactions and immune defense; HSV-1 infection induces CLU expression	Lambert et al. (2009), Carter (2010b, 2011)
Complement receptor 1 (CR1)	Homolog to glycoprotein C; promote viral entry and intracellular transport; participate into the complement system interactions and immune defense	Linard et al. (2021), Carter (2010a,b, 2011), Harris and Harris (2018)
Phosphatidylinositol-binding clathrin assembly protein (PICALM)	Facilitate viral entry, cell-to-cell transmission, and nuclear egress	Carter (2010a,b, 2011), Harris and Harris (2018)
ATP cassette transporter (ABCA7)	Induce viral reactivation; increase the efflux of sphingomyelin building viral membrane	Lambert et al. (2009), Carter (2011)
CD2-associated protein (CD2AP)	Inhibit viral infection	Carter (2011), Licastro et al. (2011)
Cluster of differentiation 33 (CD33)	Promote viral entry	Lambert et al. (2009), Carter (2011), Teuton and Brandt (2007)
Bridging integrator 1 (BIN1)	Bind to dynamin that is important for HSV-1 entry and intracellular trafficking; participate into phagocytosis in macrophages; associated with autophagy	Carter (2011), Albecka et al. (2016)
Ephrin A1 (EPHA1)	Increase brain susceptibility to viral infection	Lambert et al. (2009), Licastro et al. (2011)
β-APP Cleaving Enzyme-1 (BACE1)	HSV-1 infection upregulates BACE1 expression to promote Aβ accumulation, sequence homolog to HSV-1 genome	Carter (2010a), Wozniak et al. (2007), De Chiara et al. (2010)
β-APP Cleaving Enzyme-2 (BACE2)	Sequence homolog to HSV-1 genome	Carter (2010a)
Triggering receptor expressed on myeloid cells-2 (TREM2)	Identified as an anti-inflammatory receptor to inhibit viral infection	Zhu et al. (2020), Wu et al. (2021), Stefanie et al. (2023)

## 3. HSV-1 induces amyloid β and tau pathogenesis

The initial observation implicating HSV-1 in AD pathogenesis was the frequent detection of the HSV-1 genome in the brain of AD patients, although it was also present in some controls. To further determine the role of HSV-1 in AD, studies involving cell cultures and animal models were routinely performed to determine whether and how HSV-1 actively contributes to AD pathogenesis. One of the well-accepted mechanisms is that Aβ functions as an antiviral peptide and is induced by HSV-1 infection (Soscia et al., 2010; Eimer et al., 2018a). The colocalization of Aβ plaques and viral proteins (e.g., glycoprotein B) is observed *via* immunostaining with antibodies against HSV-1 and Aβ (Eimer et al., 2018b; Ezzat et al., 2019). To exert its antiviral activity, Aβ deposits on HSV-1 virion particles such that HSV-1 induces the oligomerization and fibrilization of Aβ through physical interaction with its surface glycoprotein B, a ligand mediating HSV-1 entry (Bourgade et al., 2022; Yirün et al., 2023). Recently, the mechanistic connections between HSV-1 and Aβ pathology have been extensively studied, and these findings collectively support a causative role of HSV-1 in the neurodegeneration of AD. Specifically, HSV-1 was shown to directly induce the accumulation of Aβ_42_ even at low multiplicity of infection (MOI = 0.3) in the neuronal cells and human induced pluripotent stem cells (hiPSCs) derived from healthy subjects (Wozniak et al., 2011; Abrahamson et al., 2021). Furthermore, increased intracellular Ca^2+^ was observed in the HSV-1-infected rat cortical neurons, leading to the Ca^2+^-dependent phosphorylation of APP and intracellular accumulation of Aβ_42_ (Piacentini et al., 2011). Albaret et al. (2023) established a model of murine neuronal cells for HSV-1 infection and determined a caspase-dependent production of Aβ_42_ oligomers. These Aβ_42_ oligomers formed aggresomes with caspase 3A, impairing neuronal apoptosis and fueling Aβ_42_ accumulation. However, in the study using 3D brain organoids of hiPSC infected with HSV-1, Aβ_42_ was more abundantly found in bystander uninfected cells (Abrahamson et al., 2021), suggesting that the interaction between HSV-1 and the host cell is more complicated and likely associated with other cellular events, such as cell-to-cell communication and signal transduction. Although varying in distinct experimental settings, these studies nevertheless uncover a plethora of roles that HSV-1 can play in AD pathogenesis and suggest that more physiological models are direly needed to dissect the molecular pathogenesis of HSV-1-induced neurodegeneration.

Neurofibrillary tangles, commonly known as tauopathy, is another pathological feature of neurodegenerative AD and strongly correlates with the progression to late stages of the disease (Iqbal et al., 2005). Tau hyperphosphorylation is the known culprit of tauopathy (Martin et al., 2013). Studies using monolayer cell culture, 3D bioengineered brain model and rodent animal models demonstrated the induction of tau phosphorylation in response to HSV-1 infection (de Chiara et al., 2019; Cairns et al., 2020; Powell-Doherty et al., 2020). Multiple tau phosphorylation sites were identified upon HSV-1 infection in neuronal cells, including serine 202, threonine 212, serine 214, serine 396 and serine 404. Kinases such as glycogen synthase kinase 3β (GSK3β) and protein kinase A were reported to mediate these phosphorylations (Wozniak et al., 2009). Later, Alvarez et al. (2012) observed hyperphosphorylated tau in the nucleus of HSV-1-infected neuronal cells, and phosphorylated tau was associated with viral DNA replication. The re-distribution and phosphorylation of tau induced by HSV-1 increased host DNA damage (Sait et al., 2021), which contributes to the neuronal cell death and potentially accelerates AD progression. In addition, anti-herpetic drugs, as alternative treatment strategy, were used in the laboratory and clinical settings to reduce the development of AD, supporting the active roles of HSV-1 in AD pathogenesis. For example, antiviral agent acyclovir (ACV) ameliorates tauopathy by inhibiting HSV-1 DNA replication (Wozniak et al., 2011). Besides the cellular kinases such as GSK3β that are activated by HSV-1 to phosphorylate Tau, HSV-1-encode kinases, i.e., serine/threonine-protein kinase UL13 and US3, can directly phosphorylate tau to its hyperphosphorylation form, further exacerbating tauopathy and neurodegeneration (Benetti and Roizman, 2004; Devanand, 2018). Future studies are required to test a hypothesis determine whether there are direct interactions between other viral factors and tauopathies in AD pathogenesis associated with HSV-1 infection. In addition, *in vivo* studies using tau mouse models to assess the roles of HSV-1 infection in the tauopathy are missing and beg for immediate investigation.

## 4. Inflammation can fend off HSV-1 infection or exacerbate HSV-1-induced neurodegeneration

**Figure 2:**
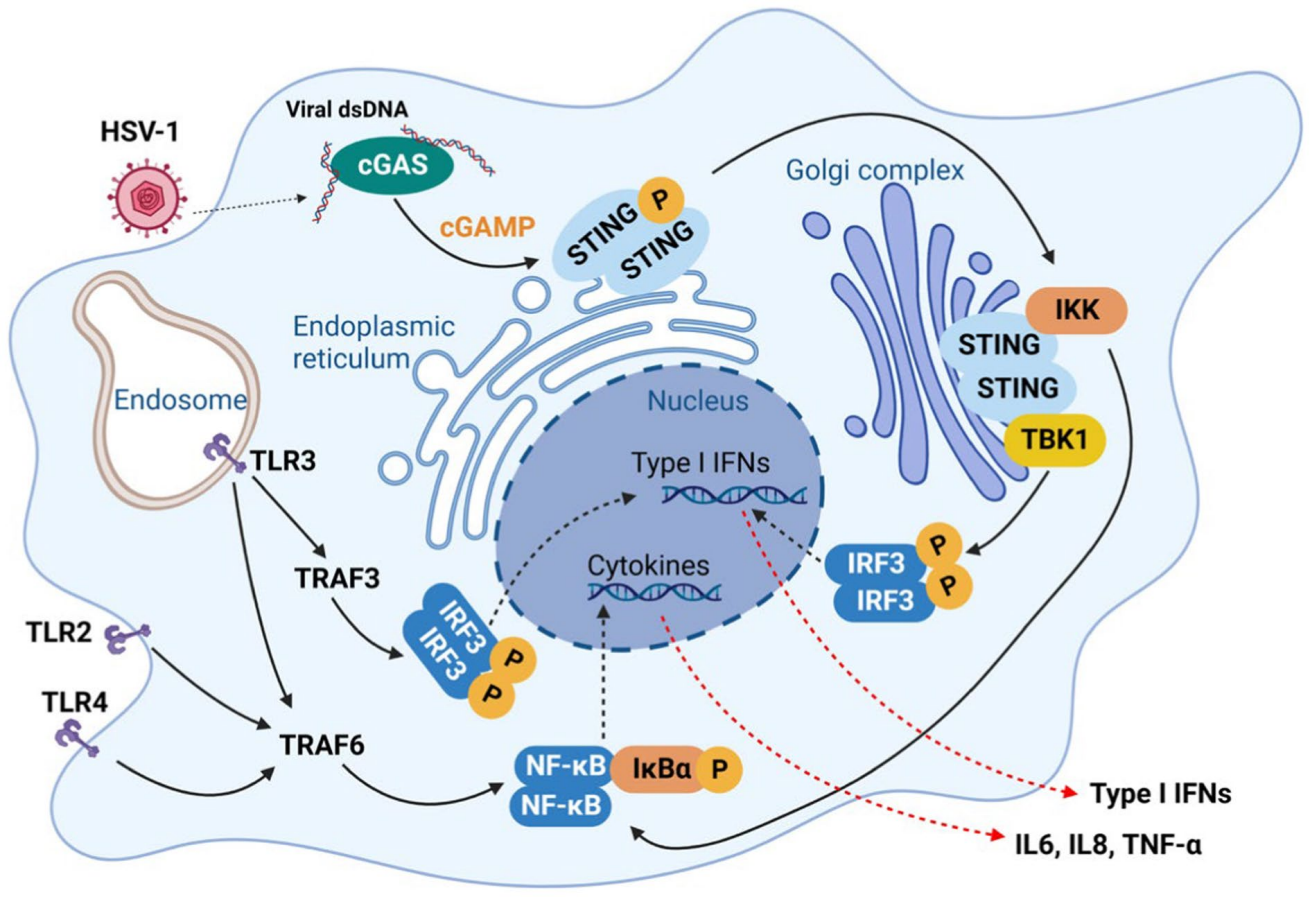
Innate immune response against HSV-1 infection in microglia. In response to HSV-1 infection, pattern recognition receptors (PRRs) which recognize the pathogen-associated molecular patterns (PAMPs), initiate series of signaling events that defend host against infecting viruses, which culminate in the production of type I interferons (IFN-I) and inflammatory cytokines, including IL6, IL8 TNF-α.

In response to viral invasion in the central nervous system (CNS), pattern recognition receptors (PRRs) recognize the pathogen-associated molecular patterns (PAMPs) and initiate series of signaling events that culminate in the production of type I interferons (IFN-I) and inflammatory cytokines to establish a potent antiviral state. In mouse models with recurrent HSV-1 infection, augmented neuroinflammatory markers, including astrogliosis, IL1β and IL6, were observed to correlate with accumulation of amyloid-β protein and tau hyperphosphorylation (de Chiara et al., 2019). The innate immune response induced by acute HSV-1 infection in the brain is considered neuroprotective in normal brain. Upon HSV-1 infection in microglia, resident macrophages of the CNS, the cyclic GMP-AMP synthase (cGAS) binds to viral dsDNA and catalyzes the synthesis of cGAMP that in turn binds to and activates the adaptor protein stimulator of IFN-I genes (STING). Activated STING travels through the ER, trans-Golgi network (TGN) and endosomes, which activates IRF3 in a TBK1-dependent manner to stimulate IFN and inflammatory cytokine production (Figure 2; Reinert et al., 2016; Fang et al., 2023; Qin et al., 2023) To assess the role of cGAS-STING pathway in HSV-1 infection, Reinert et al. (2016) used cGAS- and STING-deficient mice and found that these mice were highly susceptible to HSV-1 infection and succumbed to herpes simplex encephalitis (HSE). These findings support the protective role of innate immune response against HSV-1 infection.

Triggering receptor expressed on myeloid cells 2 (TREM2) is a transmembrane receptor strictly expressed on microglia in the brain, and maintains microglial homeostasis in metabolism and immune function (Ulland and Colonna, 2018). Variants in the TREM2 gene, exemplified by the R47H mutant, have significant association with the progression of LOAD (Guerreiro et al., 2012; Jiang et al., 2013). Several studies have demonstrated that TREM2 is an anti-inflammatory receptor against microbial infection, such as SARS-CoV-2 and HSV-1 (Zhu et al., 2020; Wu et al., 2021). Elevated viral replication was observed in TREM2-depleted co-cultures and *Trem2^−/−^* mice (Stefanie et al., 2023). Depletion of TREM2 in microglia, differentiated from iPSC, reduced innate immune response, indicated by the lower expression of *IFNB, IL6, TNFA, and MX1* induced by HSV-1 infection (Stefanie et al., 2023). Similarly, Toll-like receptor 3 (TLR3), a transmembrane receptor recognizing the dsRNA produced during HSV-1 replication, can effectively limit HSV-1 replication in CNS *via* activating the expression of *IFNB* mRNA and secretion of IFNβ (Gao et al., 2021). A cell culture study using cortical neurons derived from iPSC of HSV-1 encephalitis patients, also showed that the TLR3-deficient cells failed to restrict HSV-1 replication due to the impaired TLR3-IFN immunity (Lafaille et al., 2012). Other TLRs, such as TLR2 and TLR4, also respond to HSV-1 infection in astrocytes, resulting in the upregulation of IRF3, IRF7, IFNβ and the pro-inflammatory cytokine IL6 (Villalba et al., 2012). Findings from these *in vivo* and *in vitro* studies highlight the critical role of innate immune defense downstream of TLRs in the brain against HSV-1 infection.

Prolonged inflammation in the CNS can be pathological by creating excessive cellular stress and cytotoxicity, particularly in the AD brain (Kim and Joh, 2006; Zheng et al., 2016; Blank and Prinz, 2017; Kinney et al., 2018; Burgaletto et al., 2020). Constitutive activation of the cGAS-STING pathway in human AD and aged mice were observed (Gulen et al., 2023), and *cgas* deficient 5xFAD (*cgas*^−/−^; 5xFAD) mice presented a better cognitive behavior and reduced amyloid pathology (Xie et al., 2023). Consistently, Roy et al. (2020, 2022) determined the constitutively expressed IFN-I in the brain of AD mouse model, in which active IFN-I signaling induced synapse loss, leading to cognitive impairment. In the study using AD mouse model lacking IFN-I signaling (APP_SWE_/PS1_ΔE9_ x IFNAR1^−/−^), Taylor and Crack’s research team confirmed that the loss of IFN-I signaling conferred a predominantly anti-inflammatory glial phenotype, which protects mice from cognitive decline (Minter et al., 2016). Similarly, elevated expression of TLR2 and TLR4 in peripheral blood mononuclear cells (PBMCs) was found in AD patients compared to healthy control subjects (Zhang et al., 2012), and sustained TLR2 activation has been suggested to positively contribute to the neuroinflammation and Aβ accumulation (McDonald et al., 2016). HSV-1 reactivation in the mouse brain can increase the production of interleukin 1β (IL-1β), resulting in synaptic dysfunction through the epigenetic MeCP2-HDAC4 complex (Li Puma et al., 2023). These findings strongly support the conclusion that the innate immune activation and subsequent chronic neuroinflammation exacerbate AD pathogenesis (Heneka et al., 2015b; Zheng et al., 2016; Taylor et al., 2018).

Taken together, inflammatory response can be pleiotropic and may exert adverse effects on the brain. As Jin et al. (2021) reported that the PQBP1-cGAS-STING pathway is likely shared by viral infection and that extrinsic tau 3R/4R proteins induce microglia-mediated neuroinflammation toward neurodegenerative disease. Therefore, in the context of periodic HSV-1 reactivation in the elderly, HSV-1 infection could persistently activate PRRs (TRLs, RLRs and cGAS) (Figure 2), leading to chronic neuroinflammatory response and gradual neuron loss. Conceivably, better understanding in the mechanism of how HSV-1 induces inflammation in neurodegeneration will enable neuroprotective strategies *via* therapeutically modulating inflammation in the brain.

## 5. HSV-1 infection reprograms host metabolism to accelerate AD progression

Metabolic dysregulation is another hallmark of LOAD (Salminen et al., 2015; McAvoy and Kawamata, 2019; Fairley et al., 2021). Metabolism is a central cellular activity that orchestrates diverse fundamental biological processes, such as synaptic communication and immune response. Mechanisms by which microbes reprogram cellular metabolism have been long studied, particularly those obligate intracellular parasites such as HSV-1 (Zhang et al., 2018; Polcicova et al., 2020). Upon *de novo* infection or reactivation, HSV-1 re-directs cellular metabolism to support virus replication, such as viral genome replication, structure protein synthesis, and lipid synthesis. To date, we have learned distinct strategies that HSV-1 evolved to thwart host immune response. However, how HSV-1 and other neurotropic viruses manipulate cellular metabolism in the brain is poorly understood, not mentioning *in vivo* studies using AD mouse models. Thus, how viral infection disrupts brain metabolic homeostasis in the context of AD and AD-related dementia (ADRD) deserves further investigation.

### 5.1. Glycolysis and oxidative phosphorylation

A robust correlation between AD and mitochondria metabolic dysfunction has been established by both basic research and clinical studies (Heneka et al., 2015a; Fairley et al., 2021). In AD, a number of mitochondrial abnormalities have been identified, such as structural alterations, age-dependent accumulation of oxidized mitochondrial DNA (mtDNA), loss of mitochondrial membrane potential, excessive mitochondrial ROS production, diminished mitochondrial adenosine triphosphate (ATP), disrupted electron transport chain (ETC), and imbalanced mitochondrial fragmentation and fusion, which act alone or in combination to impede mitophagy in microglia and other brain cells (Cadonic et al., 2016; Rajmohan and Reddy, 2017; Pradeepkiran and Reddy, 2020; John and Reddy, 2021; Morton et al., 2021; Lee et al., 2022). Additionally, chronic exposure to Aβ and phosphorylated Tau (p-Tau) proteins derails the expression of LOAD-associated genes, including *Cst7, Igf1, Apoe, Spp1, Trem2, Lgals3* (Grubman et al., 2021), thereby inducing mitochondrial toxicity and metabolic dysfunction in microglia by downregulating the AKT-mTOR-HIF1a pathway (Baik et al., 2019).

**Figure 3:**
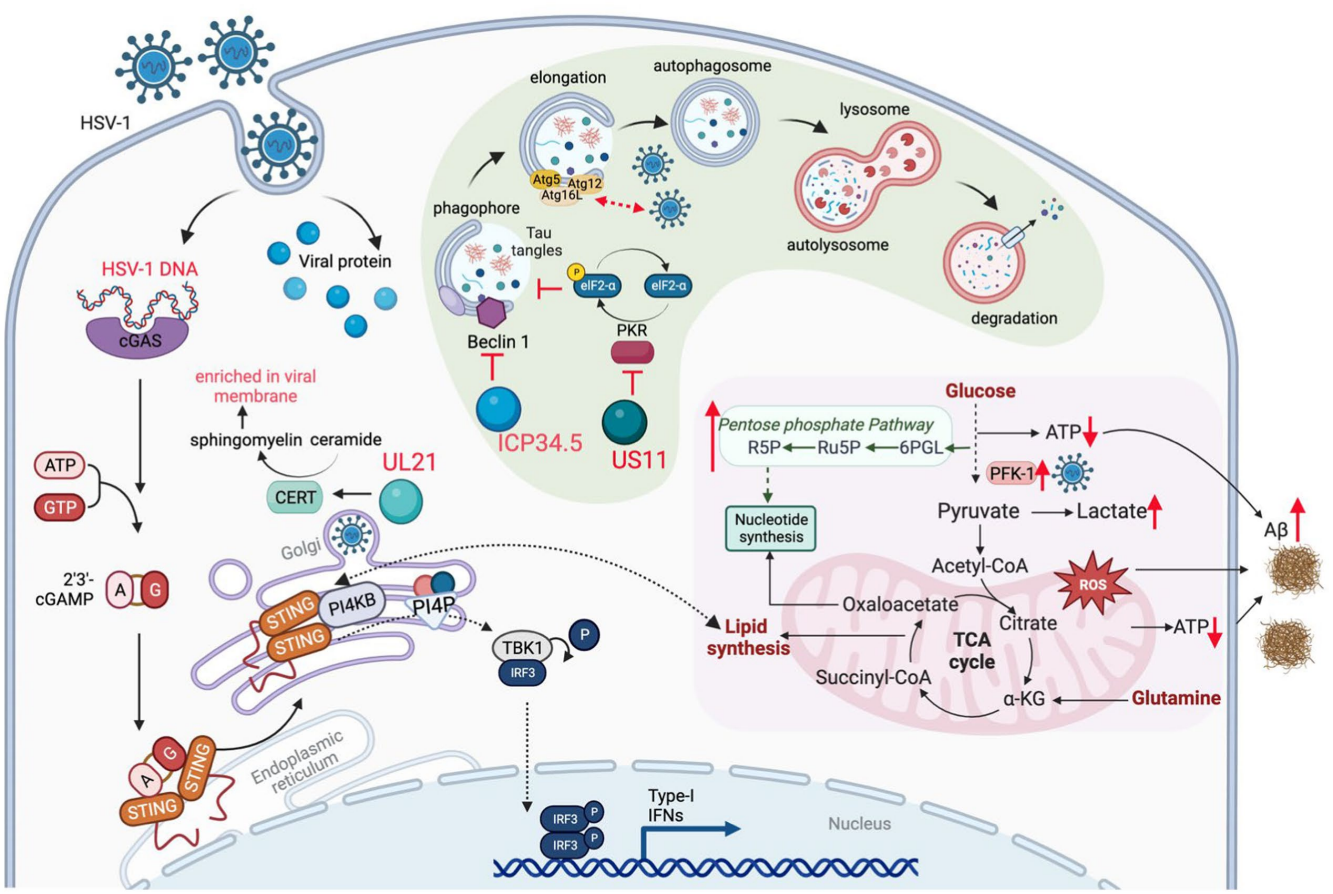
HSV-1 infection induces metabolic dysfunction to accelerate AD progression. HSV-1 infection reprograms carbon metabolism skewed toward aerobic glycolysis that supplies glycolytic intermediates crucial for viral replication, and, to a lesser extent, relying on oxidative phosphorylation that produces reactive oxygen species (ROS) and limited energy (ATP). In addition, innate immune response (e.g., via the cGAS-STING pathway) triggered by HSV-1 also alters lipid metabolism, which contributes to the brain degeneration. To counteract viral protein degradation through autophagy, HSV-1 deploys several viral proteins (e.g., ICP34.5 and US11) to inhibit autophagy pathway, which disrupts the degradation of misfolded proteins, such as Aβ fibrils. Therefore, HSV-1 infection damages brain metabolic homeostasis leading to the development of AD.

One of the most important functions of mitochondria is oxidative phosphorylation (OXPHOS) *via* the tricarboxylic acid (TCA) cycle, providing the energy source to maintain most ATP-dependent biological processes of a cell. Neurons are highly dependent on the mitochondrial OXPHOS to provide energy for their synaptic functions. In highly proliferating cells, aerobic glycolysis however produces diverse metabolites that are funneled to synthesize key cellular building blocks. Upon infection, HSV-1 reprograms cellular metabolism to aerobic glycolysis that supplies metabolites crucial for viral replication. HSV-1-induced aerobic glycolysis increases the production of glucose-6-phosphate and fructose-1,6-bisphosphate to fuel the pentose phosphate pathway (PPP) (Figure 3; Vastag et al., 2011). The PPP and serine synthesis pathway (SSP) yield ribose-5-phosphate and one-carbon unit, respectively, for *de novo* nucleotide synthesis (Vastag et al., 2011). Thus, HSV-1 infection imposes a metabolic switch from mitochondrial oxidative phosphorylation to aerobic glycolysis, which undermines the proper functions of neurons. This dysregulated metabolism, characterized by increased glucose consumption, inefficient ATP production and excessive lactate secretion, has been observed as a prevalent feature in the early stages of AD (Crane et al., 2013; Croteau et al., 2018; Gordon et al., 2018). However, a possible causative role of HSV-1-induced glycolysis in AD pathogenesis has not been reported. Large-scale proteomic studies also found that protein network modules related to glucose metabolism are key pathogenic factors associated with AD pathology and cognitive impairment (Johnson et al., 2020; Vaillant-Beuchot et al., 2021). The role of these metabolic proteins in HSV-1 infection remains largely unexplored. Gene expression studies have shown significant downregulation of transcripts of proteins involved in the glycolysis, TCA cycle, OXPHOS, and related pathways in post-mortem hippocampal samples of AD patients (Brooks et al., 2007; Sorrentino et al., 2017). Interestingly, HSV-1 infection increased OXPHOS, glucose consumption, and lactate production by upregulating phosphofructose kinase M (PFKM, PFK-1) activity, suggesting the simultaneous activation of the aerobic glycolysis and OXPHOS (Abrantes et al., 1822). Currently, it is not clear how HSV-1 achieves this metabolic reprogramming and how such skewed metabolic activity shapes the course of AD pathogenesis.

Recurrent HSV-1 infection induces oxidative damage (Protto et al., 2020) that is linked to AD-related pathophysiology, including dysregulation of calcium homeostasis, Aβ accumulation, tau hyperphosphorylation, synaptic dysfunction, neuroinflammation and neurodegeneration (Zhao and Zhao, 2013; Tönnies and Trushina, 2017). For example, pseudorabies virus (PRV) and HSV-1 infection disrupt mitochondrial dynamics and motility in cultured neurons, by increasing the intracellular Ca^2+^ that further impinges on the mitochondrial membrane potential (Kramer and Enquist, 2012). HSV-1 infections can also increase the generation of reactive oxygen species (ROS), likely resulting from elevated mitochondrial OXPHOS due to metabolic activation or inflammatory response. Excessive ROS can cause pathogen-associated proteins to induce pathological changes in neural tissue, leading to chronic brain inflammation that is often observed in prototype neurodegenerative diseases (Limongi and Baldelli, 2016). Previous studies have shown that HSV-1 infection elevates ROS levels in mouse microglial cells and disrupts mitochondrial transport in neurons (Kramer and Enquist, 2012). In HSV-1-infected human neuronal cells, oxidative stress decreases Aβ secretion and enhances intracellular Aβ accumulation (Figure 3), while significantly impairing autophagy (Santana et al., 2013). HSV-1 infection and oxidative stress also hinder lysosomal function, a phenomenon observed early in AD development (Kristen et al., 2018). A recent study revealed that acute infection with HHV-6A and HHV-6B also inhibits autophagy and increases endoplasmic reticulum stress (Romeo et al., 2019), likely through the same mechanism observed in HSV-1 infection (O’Connell and Liang, 2016). Impaired autophagy has been identified in the AD brain but not in normal brains (Boland et al., 2008). These studies suggest that oxidative stress, autophagic dysfunction, and impaired lysosome system in HSV-infected cells may result in inefficient clearance of Aβ amyloids, thereby directly fueling Aβ accumulation in the AD brain.

The intricate relationship between mitochondrial dysfunction, AD and HSV-1 infection has been the focus of recent research, providing new insights into the pathogenesis of AD. Mitochondrial dysfunction accounts for the energy impairment, oxidative stress, and neurodegeneration observed in AD, while HSV-1 infection further exacerbates these pathological processes. Future research on the interplay between mitochondrial dysfunction, HSV-1 infection, and AD pathogenesis will surely yield molecular information concerning HSV-1 in mitochondrial homeostasis and neurodegeneration. This knowledge could pave the way for the development of novel therapeutic strategies targeting mitochondrial dysfunction and HSV-1 infection to improve the prognosis of neurodegeneration and the quality of life of individuals with AD. Interdisciplinary fertilization crossing the field of neurodegeneration, virology and mitochondrial biology can greatly advance our understanding of molecular events underpinning neurodegeneration and exposing key molecules for therapeutic development.

### 5.2. Lipid metabolism

As an obligate intracellular pathogen, HSV-1 relies on the cellular machinery to synthesize nucleic acids, amino acids, and complex lipids that are building blocks of the virion progeny. Thus, HSV-1 has evolved diverse mechanisms to redirect cellular metabolic activity toward viral macromolecule synthesis. This reprogram can be achieved directly *via* viral metabolic enzymes, such as thymidine kinase, dUTPase, uracil-DNA glycosylase, and ribonucleotide reductase that participate in key steps of the nucleotide metabolism (Thaker et al., 2019). Alternatively, *via* regulating cellular proteins, HSV-1 alters the metabolic pathways that provide essential intermediates for viral replication. Argininosuccinate synthetase 1 was down-regulated by HSV-1 infection, which redirects aspartate for *de novo* nucleotide synthesis (Grady et al., 2013). HSV-1 UL21-mediated dephosphorylation activates cellular ceramide transport protein (CERT) and facilitates the synthesis of sphingomyelin from ceramide. Sphingomyelin, enriched in viral membrane, is an essential component of the trafficking vesicles between trans-Golgi network (TGN) and the plasma membrane, a vehicle crucial for neurotransmitter secretion and release (Figure 3; Benedyk et al., 2022). Moreover, recent studies have revealed that innate immune activation and lipid metabolism are intimately coupled during HSV-1 infection. It is well established that HSV-1 activates the cGAS-STING DNA-sensing pathway that mounts the antiviral inflammatory response. In addition to a well-defined inflammatory response (such as IFN induction), activated STING also promotes the fatty acid desaturase 2 (FADS2)-mediated desaturation to increase the synthesis of poly-unsaturated fatty acid (PUFA). To do that, activated STING directly inhibits the activity of the FADS2-associated delta-6 desaturase (D6D) (Vila et al., 2022). Intriguingly, PUFA can inhibit STING-mediated signaling, likely contributing to the resolution of inflammation downstream of STING. It is also possible that HSV-1 elevates the PUFA levels to negate STING-dependent innate immune defense. Recently, Fang et al. (2023) demonstrated that the PI4KB-synthesized PI4P is required for STING activation and downstream signal transduction, further strengthening the crosstalk between lipid metabolism and innate immune response (Figure 3). These findings imply that HSV-1 infection activates innate immune response that in turn modulates host lipid metabolism, and that immune response and lipid metabolism may differentially influence the amplitude and duration of the immune response. How the crosstalk between lipid metabolism and innate immune activation downstream of STING contributes to neuroinflammation in the AD brain is not clear. Although recent studies have highlighted the antiviral activity of the cGAS-STING pathway against HSV-1 in the brain, the chronic activation of the cGAS-STING pathway is responsible for AD pathogenesis in mouse models (Xie et al., 2023).

As described above, APOE4, a primary genetic risk factor of AD, is responsible for binding cholesterol-laden lipid and can increase lipid accumulation in the blood stream (Huang and Mahley, 2014). Expressing APOE4 in human induced pluripotent stem cell (iPSC)–derived astrocytes and glia cells alters lipid homeostasis by elevating unsaturated fatty acids and accumulating intracellular lipid droplets (Sienski et al., 2021). Accumulation of intracellular cholesteryl esters upregulated the phosphorylated Tau (p-Tau) by inhibiting the proteasome function in iPSC-derived neurons of AD patients (van der Kant et al., 2019). Moreover, the level of lipid is correlated with the strength of inflammatory response (Derk et al., 2018), as further validated by a study demonstrating that STING activation alters lipid metabolism (Vila et al., 2022). Collectively, these results thus concluded that the lipid metabolism, immune response and HSV-1 infection are intimately connected, thus calling for further studies to determine how HSV-1 infection induces immune response and alters lipid metabolism to impact AD pathogenesis.

### 5.3. Autophagy

Autophagy is an evolutionarily conserved cellular process that degrades and recycles undesired cellular constituents and cytoplasmic compartments under nutrient-constrained conditions (Liang, 2010). Though tightly regulated, autophagy is critical for the clearance of aggregated proteins that undermine the function of the brain of several neurodegenerative diseases, including AD, amyotrophic lateral sclerosis and familial Parkinson’s disease (Nixon, 2013). The inhibition of autophagy in neurons of the brain leads to the accumulation of amyloid proteins and phosphorylated Tau, triggering the neuronal cell death and degeneration of the brain of AD patients. In addition to digesting intracellular aggregated proteins or dysfunctional organelles such as mitochondria, autophagy serves as a host defense mechanism against invading microbes. It is not surprising that viruses have evolved multiple strategies to deflect autophagy, which promotes viral replication or persistent infection. For example, HSV-1 ICP34.5 interacts with autophagy protein Beclin 1 to inhibit cellular autophagy in fibroblasts and neurons, contributing to its fatal encephalitis (Orvedahl et al., 2007). Independent of Beclin 1 and mTOR, HSV-1 US11 impedes autophagy via its binding to PKR, an RNA-activated protein kinase that induces autophagy *via* eIF2α phosphorylation and translational shutdown (Figure 3; Lussignol et al., 2013). Recently, Bearer group analyzed the autophagy genes (ATG) through multiple publicly available genetic datasets of AD cohorts, and found that decreased ATG gene expression is correlated with higher viral load and occurrence of cognitive impairment (Nafchi et al., 2022). These findings suggest a causative role of HSV-1 infection in autophagy dysfunction that may fuel AD pathogenesis.

## 6. Conclusion and future perspectives

The role of HSV-1 in AD pathogenesis is emerging and is supported by mounting evidence from diverse studies. Yet, how exactly HSV-1 infection affects cellular processes that differentially impact neurodegeneration underpinning AD is largely unknown, despite the antimicrobial peptide function of Aβ. Here, we reviewed the recent advances in molecular studies on how HSV-1 infection alters key cellular activities that are relevant to AD pathogenesis. HSV-1 may provide a useful tool to probe the molecular mechanism of neurodegeneration and offers answers toward effective therapy.

1. Viruses, particularly neurotropic viruses, are powerful tools for neuroscience and related studies. If we understand the intimate interactions between viruses and the host with great detail at the molecular level, therapeutic inventions using engineered self-attenuated viruses can be properly designed toward the diagnosis, prevention, and effective treatment of AD.
2. Although many studies addressed the mechanism of HSV-1-induced damage in cultured cells and inbred animals, the difference among distinct cell types of the brain and impact on cell-to-cell communications are less understood. Systematic investigation and integration of results from different cell types are imperative to unravel the biological significance of HSV-1 infection in the physiological setting of the brain.
3. Immunometabolism is an emerging inter-discipline, with a significant impact across many fields of biology research, while imminent research questions regarding AD pathogenesis yet to be answered. Molecular link between metabolism and immune response to elucidate the dual roles of metabolic enzymes and immune signaling molecules during HSV-1 infection begs for further investigation.

## Author contributions

SF and PF conceived of the review. SF, YZ, ZS, and ZC: draw figures. SF, YL, YZ, ZS, and PF wrote the manuscript. All authors commented on and approved the manuscript.

## Funding

The Feng research group is supported by AG070904 and CA285192 from NIH, and microbial pathogenesis in AD grant from IDSA. CB research team is supported by Alfred E. Mann Family Foundation.

## Conflict of interest

CB is the chief scientific advisor of ChromaDex and co-founder of Alphina Therapeutics. PF is a consultant for Marc J Bern & Partners LLP.

The remaining authors declare that the research was conducted in the absence of any commercial or financial relationships that could be construed as a potential conflict of interest.

## Publisher’s note

All claims expressed in this article are solely those of the authors and do not necessarily represent those of their affiliated organizations, or those of the publisher, the editors and the reviewers. Any product that may be evaluated in this article, or claim that may be made by its manufacturer, is not guaranteed or endorsed by the publisher.
